# Sexual Selection and the Treatment of Predecessors’ Progeny by Replacement Mates

**DOI:** 10.3389/fpsyg.2022.924238

**Published:** 2022-06-13

**Authors:** Martin Daly, Gretchen Perry

**Affiliations:** ^1^Department of Psychology, Neuroscience & Behavior, McMaster University, Hamilton, ON, Canada; ^2^Department of Sociology, Anthropology & Human Services, University of Canterbury, Christchurch, New Zealand

**Keywords:** Cinderella effects, mating effort, parental investment, sexual selection, stepchild, stepparent

## Abstract

Darwin’s theory of sexual selection provides a useful framework for understanding the behavior of stepparents. A non-human animal whose new mate has dependent young may kill, ignore, or adopt the predecessor’s progeny. The third option has been interpreted as courtship (“mating effort”), and whether selection favors such investment over killing or ignoring the young apparently depends on aspects of the species-typical ecology and demography. The tripartite categorization of responses is a simplification, however, There is variability both within and between species along a continuum from rejection to “full adoption.” The average stepparent invests less than the average birth parent, but more than nothing. Human stepparents have often been found to kill young children at higher rates than birth parents, but stepparental infanticide cannot plausibly be interpreted as a human adaptation, both because it is extremely rare and because it is almost certainly more likely to reduce the killer’s fitness than to raise it. How sexual selection theory remains relevant to human stepparenting is by suggesting testable hypotheses about predictors of the variability in stepparental investment.

## Introduction

Darwin’s (1871) theory of “sexual selection” initially drew a much cooler response than had been the case with his great treatise on “natural selection” 12 years previously. Even biologists who were totally on board with the earlier theory, including its co-discoverer Alfred Russel Wallace, were skeptical (Richards, 2017). Many thinkers who were prepared to accept the proposition that differential efficacy in coping with extrinsic environmental factors could give direction to the evolution of phenotypes nevertheless balked at the idea that intra-specific interactions could play a similar selective role (Gayon, 2010).

A hundred years later, Darwin’s second great theory had still inspired little further theorizing apart from Fisher’s (1915; 1930) theory of the “runaway” process, and scarcely more empirical work. Then research on sexual selection suddenly blossomed, and in the 1970s and 1980s, many hundreds of studies of mating competition and mate choice were published (see e.g., Thornhill and Alcock, 1983; Eberhard, 1985; Andersson, 1994), vastly more, in fact, than the number of studies of natural selection by the extra-specific environment (Endler, 1986).

An important stimulus for this belated blossoming was the publication of *Sexual Selection and the Descent of Man, 1871–1971*, an edited volume celebrating the centenary of Darwin’s opus (Campbell, 1972). This compendium included chapters by such luminaries as Loren Eiseley, George Gaylord Simpson, Theodosius Dobzhansky, and Ernst Mayr, but in a review in *Science*, George Williams (1973) proposed that “Parental investment and sexual selection” by the relatively unknown Robert Trivers (1972) was one of two chapters that would turn out to be “the most permanently valuable part of the book” (p. 788). In this prediction, Williams was, as usual, prescient. In the ensuing half century, Trivers’s richly detailed argument that differential “parental investment” is the key to understanding the operation and outcomes of sexual selection has undeniably had the greatest influence on both research and further theorizing (Andersson, 1994; Mogilski, 2021).

## Sexually Selected Infanticide

Sarah Blaffer Hrdy was a graduate student pursuing primatological research in India when the new enthusiasm for sexual selection was sweeping through animal behavior. Trivers was a member of her supervisory committee and visited her at her field site in Abu, Rajasthan. Her first empirical publication (Hrdy, 1974) was about “male-male competition and infanticide” in the Hanuman langurs she studied, and in her acknowledgments, she credited the “inspirational teaching of R.L. Trivers, whose ideas are fundamental to this paper” (p. 55).

A one-male, multi-female troop structure was characteristic of Hrdy’s monkey subjects, and bachelor males regularly tried to overthrow current resident breeders. Whenever one of these “political changes” succeeded, Hrdy observed that nursing infants in the troop soon disappeared, and on a few occasions, she or a local informant directly observed a new male’s fatal attack. Inferring that such attacks probably caused the unwitnessed deaths as well, Hrdy advanced an explanation in terms of sexual selection: Under conditions like those prevailing at Abu, “infanticide might permit an incoming male to use his short reign over a troop more efficiently. By eliminating an infant unlikely to be his own, a newcomer could hasten the mother’s return to sexual receptivity so that she could then bear his own offspring” (p. 46). Although she clearly favored this hypothesis, and bolstered it with an abundance of observational and circumstantial evidence, she also identified areas where further evidence would be needed to conclusively dispose of the alternative possibility that infanticide was just a non-adaptive byproduct of something else.

Early reaction to Hrdy’s argument was muted and mixed, but she inspired some researchers (e.g., Bertram, 1975), and she soon captured the attention of a larger audience with an article (Hrdy, 1977) in the *American Scientist*, a semi-popular magazine whose subscribers, in a pre-internet age, included many behavioral biologists. Here, she was no longer so cautious: Her title, in a large bold font, was “Infanticide as a Primate Reproductive Strategy” and a subheading, also in an over-sized font, read “Conflict is basic to all creatures that reproduce sexually, because the genotypes, and hence self-interests, of consorts are necessarily non-identical. Infanticide among langurs illustrates an extreme form of this conflict.” She clearly explained the theory while providing engaging accounts of her observations, of relevant langur natural history, and of prior observations, extending back more than a century, which had partially anticipated and now reinforced her insights. Hrdy (1979) followed up with a substantial, scholarly treatment of the theory’s applicability to a wider range of animals than just primates.

Some authors (e.g., Curtin and Dolhinow, 1978; Sussman et al., 1994) opposed the sexual selection hypothesis, arguing that infanticide was better interpreted as a maladaptive response to one or another sort of human interference, but in our view, this thesis never fit the facts very well. The timing and selectivity of infanticide, as well as an accumulating body of observational evidence that males of several species systematically stalked and calmly dispatched their victims, seemed clearly to fulfill Williams’s (1966) “design” criteria for the identification of an adaptation. In retrospect, the tenacity of those who insisted that infanticide must be pathological seems best interpreted as exemplary of “naturalistic fallacy” thinking whereby only “good” things could be “natural,” along with a dollop of the naïve group-level adaptationism that Williams (1966) had demolished, and the equally naïve anti-adaptationism that Mayr (1983) and others eventually sent packing (Alcock, 2003).

Hrdy’s sexually selected infanticide hypothesis has been widely vindicated, and no serious controversy remains about its applicability to various species in the natural world (Parmigiani and vom Saal, 1994; Ebensperger, 1998; Packer, 2000; Van Schaik and Janson, 2000; Palombit, 2012; Lukas and Huchard, 2014). Such behavior is by no means ubiquitous, however, and its distribution demands explanation.

## Kill, Ignore, or Adopt?

The best documented cases of sexually selected infanticide initially came from studies of mammals, and the perpetrators were always males. Female mammals also kill young who are not their own, but never in order to usurp the parental efforts of the fathers (Lukas and Huchard, 2019). Indeed, Hrdy’s original statement of the hypothesis, quoted above, was premised on the idea that the mammalian female’s obligate investment of time in gestation and lactation constitutes a contested limiting reproductive resource from the male perspective.

In other vertebrate classes, the situation facing the two sexes is often crucially different. Females usually oviposit shortly after their eggs are fertilized (birds), or even before (many fishes and amphibians), with the effect that major male roles in even the earliest forms of parental care can more readily evolve. One consequence has been the recurrent evolution of what is, from our mammal-centric perspective, “sex role reversal” (Eens and Pinxten, 2000; Janicke et al., 2016). In several species of Charadriiform shorebirds, for example, males incubate the eggs and guard the hatchlings alone, and in some of these, females are bigger and brighter than males, mate polyandrously, and defend a territory that incorporates the smaller territories of two or more males (Jenni, 1974). From the perspective of a female who has ousted a rival territory-holder, any male who is incubating her predecessor’s eggs is wasting time and energy, and such females sometimes set about destroying those eggs (Stephens, 1982; Emlen et al., 1989).

This sort of sex role reversal is rather rare even among birds, however (Cockburn, 2006). More than 90% of avian species are socially monogamous, and more often than not, parental care is provided by both mates. In a few such species, replacement mates of both sexes may kill their predecessors’ nestlings after pairing up with a widowed or deserted partner (e.g., Freed, 1986; Veiga, 1990; Chek and Robertson, 1991), but providing care to a new partner’s dependent young is also a common response, and simply ignoring them is another. What explains the variability? Oddly, although infanticide and “stepparental adoption” present as polar opposites, there is good reason to believe that sexual selection explains both. Does this mean that whatever we might discover can be “explained” *post hoc* by the theory of sexual selection, which is thus predictively empty? Not if the theory directs us to hypotheses about likely correlates and predictors of these alternative responses.

Rohwer (1986) was the first to articulate the problem in these terms. On this basis, he proposed several possible correlates of the alternative responses of avian “stepparents,” and then reviewed the available evidence. He began by framing the quest for adaptationist explanations as one of “asking when adoption rather than infanticide will maximize the residual reproductive value (Williams, 1966) of sexually and socially mature adults confronted with unrelated but dependent young who have lost a parent” (Rohwer, 1986: 354). Under what conditions would one response or the other elevate the replacement mate’s chances of rearing future broods with the new partner, or perhaps even with other potential partners? In a migratory songbird species with high adult mortality, a low incidence of breeding with the same partner in successive years, and a diminishing chance of successful renesting as the breeding season ticks away, a fitness payoff from stepparental care is unlikely, and such birds commonly kill. In a non-migratory species in which pair bonds may endure for years, by contrast, a widowed parent of unfledged young has more leverage to make stepparental “adoption” the price of re-partnering. More generally, relevant considerations include whether mates and/or territories are scarce, whether renesting in the same season is feasible, how long pair bonds can persist, whether territories and pair bonds are maintained or abandoned after brood failure, whether helping or infanticide or both help maintain the new mate’s residual reproductive value, and to what degree providing care to unrelated young entails lost opportunities or other costs. The evidence that Rohwer (1986) was able to muster was spotty and often anecdotal, but in general, it supported his hypothesis that the cross-species variation in the behavior of stepparents is attuned to social and ecological determinants of the fitness consequences of the alternatives.

Rohwer et al. (1999) updated the initial review’s arguments and evidence, finding further support for the hypothesis that stepparenting functions as “mating effort” in birds and in other taxa as well, and drew this conclusion: “The principal lesson of this review for students of the human animal is that investing stepparents are neither peculiar to our species nor beyond adaptationist explanation. Although stepparental infanticide occurs in many diverse taxa, it perhaps even more often is the case that non-human stepparents tolerate and care for their wards. And although many puzzling cases remain to be fully explained, it appears that stepparental tolerance and care are best interpreted as acceptable costs of courtship” (p. 386–387).

Although Rohwer (1986) initially drew a categorical distinction between “ignoring” a new partner’s offspring and “full adoption,” there are intermediate possibilities, and the Rohwer et al. (1999) review describes a number of cases that appear to occupy this middle ground. In both Western and Eastern Bluebirds, for example, stepfathers sometimes feed the young, but they do so at substantially lower rates than fathers (Meek and Robertson, 1992; Dickinson and Weathers, 1999). We suggest that this middle ground is also where the typical behavior of human stepparents falls.

## *Homo sapiens* Is a Stepparenting Species

The “mating effort” interpretation of stepparental tolerance and care seems clearly to be applicable to our own species (Anderson, 2000; Gray and Anderson, 2010). The stepparent-stepchild relationship is an ancient and cross-culturally universal element of human societies. The effects of several considerations–our species’ lengthy period of childhood and juvenile dependency, the unique degree to which people invest simultaneously in children of different ages, and appreciable incidences of both mortality and divorce during the reproductive years–have long combined to ensure that many single parents re-entered mating markets. A common recourse of widowed or divorced parents is to foster their dependents to trustworthy kin, especially grandmothers, and to then re-enter the mating market unencumbered (Rende Taylor, 2005; Scelza and Silk, 2014; Perry, 2021), but many others retain their children when they “remarry” (in the broad sense, i.e., including *de facto* unions). There is no reason to doubt that these things have been true for hundreds of generations.

The “family studies” literature is potentially misleading in these regards. Focusing solely on trends in the rich world over a few decades, many social scientists have endorsed Cherlin’s (1978) proposal that stepparenthood is a “new role” whose tensions derive from the fact that relevant norms are still being worked out. However, the incidence of stepparenthood was actually higher in recent centuries than it is today, mainly because of higher mortality in young adulthood (e.g., Dupâquier et al., 1981). For example, about a third of the female population of two 19th century cities in Netherlands had been widowed at least once by the age of menopause (Van Poppel, 1995). And whereas contemporary infants and toddlers very rarely reside with father and stepmother, this is and was much more prevalent in societies with substantial levels of maternal mortality (Warner, 2018). Perhaps most importantly, studies of contemporary foragers, who provide our best models of deep human history, often indicate that their children were substantially more likely to reside with stepfathers than children in any modern nation state (e.g., Hewlett, 1991; Hill and Hurtado, 1996; Marlowe, 1999). The adaptive problems that stepfamily formation presents have been components of human social life for many millenia, and the psychology of a nuanced response to the demands of children who are not one’s own has furthermore evolved in a highly social context of alloparental care by other interested parties as well as stepparents (Burkart et al., 2009).

There is abundant evidence that the feelings and behavior of human stepparents differ from those of birth parents in ways that are consistent with the view that stepparents are more restrained in their willingness to invest (reviews by Daly and Wilson, 1988, 1994b,^1996^, ^1998^, ^2008^). Examples include differential expressed affection (e.g., Duberman, 1975), the greater prominence of child support as a source of marital strife in stepfamilies than in birth families (e.g., Messinger, 1976), and differential treatment in domains ranging from hostile interactions (e.g., Flinn, 1988) and vigilance (e.g., Tooley et al., 2006) through ensuring children’s medical and dental health (Case and Paxson, 2001) to financial support (e.g., Zvoch, 1999), bequests (e.g., Erixson and Ohlsson, 2019), and various sorts of helping out (e.g., Anderson et al., 1999; Daly and Perry, 2021).

Daly and Wilson (1998) dubbed such manifestations of preference for birth children over stepchildren “Cinderella effects.” This label, which has caught on, was perhaps unfortunate because the fairy-tale Cinderella was the victim of a discriminative stepmother whereas most research on the phenomenon has concerned stepfather families (Daly and Wilson, 2008). That said, however, it is clear that stepparents of both sexes discriminate. Studies of US household expenditures have been particularly enlightening in this regard: Princeton economist Anne Case and collaborators have shown that children with stepmothers suffer reduced support, net of family income, in domains that are largely under female control, such as medical checkups and expenditures on food, whereas it is on “big ticket” items such as tuition fees that they are disadvantaged in stepfather households (Case et al., 2000, 2001; Case and Paxson, 2001).

## “Cinderella Effects” in Violence Against Children

The various Cinderella effects described in the preceding paragraphs were documented in samples of parents that were drawn in such a way as to be representative of their respective populations, and presumably portray average levels of discrimination by parents in general. A less frequent but extremely serious domain of stepchild disadvantage is that of child maltreatment, and it is in the most extreme forms that the largest Cinderella effects have been found. In “baby battering” cases in which shaking and/or blows to the head or abdomen by enraged caretakers were fatal, the case numbers are often indicative of a greater risk at the hands of stepfathers than birth fathers on the order of 100-fold or more. In the first published report exemplifying this contrast, Scott (1973) reported that male perpetrators charged with fatal baby battering in a jurisdiction in the south of England consisted of 14 “putative fathers” and 15 stepfathers, whereas a national survey of a 1970 birth cohort (Wadsworth et al., 1983) indicates that coresiding birth fathers would have outnumbered stepfathers by more than 100 to 1 in a population-at-large sample with the same age distribution as that of the battering victims. The most extreme example of which we are aware comes from Australia: Wallace (1986) reported that the perpetrators of fatal baby battering in New South Wales in 1968–1981 included 17 stepfathers, 11 putative genetic fathers, and one adoptive father. When Australian household survey data are used to estimate living arrangements in the population-at-large, Wallace’s data indicate a Cinderella effect of about 300-fold (Daly and Wilson, 2008). For additional examples, see Daly and Wilson (2008) and Daly (2022).

The evidence regarding lethal abuse by stepmothers is much sparser than is the case for stepfathers. Although stepmother cases exist in all large homicide data sets, small children so rarely reside with them that any estimate of a homicide rate must have huge confidence intervals. However, on the basis of analyses of national data from the FBI, Weekes-Shackelford and Shackelford (2004) were able to estimate rates indicating that US stepmothers, like stepfathers, were substantially and significantly more likely to kill young children than birth mothers. This is impressive when one considers that the birth mother cases included neonaticides, a very different sort of killings that often constitutes almost half of all filicides. However, a problem with this FBI data set is that the codes for “stepmothers” and “stepfathers” were not typically applied in cases where the killer’s partnership with the birth parent was a *de facto* marriage (Daly, 2022).

Stepmother-stepfather comparisons can be made with more confidence when we turn to non-lethal child abuse. Daly and Wilson (1981) and Creighton and Noyes (1989) analyzed large data sets of mandated abuse reports from the United States and the United Kingdom, respectively. Both data sets contained many stepmother cases, and in both, the rates of validated physical abuse in stepmother and stepfather households were roughly similar and far in excess of those in two-birth-parent households. A different sort of evidence comes from an interview study of South Korean schoolchildren, who reported identical rates of having been beaten at home in stepmother and stepfather households, both rates again far in excess of what children living with both birth parents reported (Kim and Ko, 1990). Finally, stepmother households are sometimes even more extremely overrepresented than stepfather households in the domestic circumstances of adolescent runaways who testify that they are fleeing abusive homes (e.g., Powers et al., 1990).

Oddly, although massive Cinderella effects in child homicide have been well documented in several countries, there have been relentless efforts to promote skepticism about their reality. Detailed rebuttals of some prominent examples are provided by Daly and Wilson (1998, 2001, 2008) and Daly (2022). Perhaps this “disinformation campaign” (Wilson and Daly, 1999) has been motivated by a wish to destigmatize stepparents who are doing their best in emotionally trying circumstances, but it does a disservice to the goal of establishing evidence-based child protection policies and practice (Perry and Daly, in press).

## Stepparental Ambivalence and Resentment

Can infant-killing by replacement mates be understood as a sexually selected adaptation in human beings, as it is in Hanuman langurs, lions, and many other species? Clearly not! Infanticide by human stepparents fulfills none of the criteria for identifying adaptation, as laid out by Williams (1966) and further discussed by Mayr (1983) and Andrews et al. (2002). First, in no society or situation is it routine; although the rate at which Canadian preschoolers were beaten to death by coresiding stepfathers in 1974–1990, for example, was more than 120 times the corresponding rate at the hands of coresiding birth fathers, the higher rate by stepfathers still amounts to less than one death per 3000 children at risk per annum (Daly and Wilson, 2001). Furthermore, there are no data supporting the hypothesis that killing stepchildren enhances or ever enhanced the killers’ average fitness in any human society; even in traditional societies without formal institutions of law enforcement, self-interested violence is constrained by the threat of vengeance (McCullough, 2008) and those suspected of killing even an infant risk becoming targets of the victim’s kin (e.g., Chagnon, 1988). But perhaps the most telling evidence against the hypothesis that killings of stepchildren are the direct expressions of an adaptation is that they are typically performed with spectacular inefficiency: Post-mortem examinations of victims often uncover a prior history of weeks or months of non-fatal assaults (e.g., Scott, 1973), and for every stepparent who kills, there are a great many more who inflict non-lethal damage that raises their own and their partners’ investment costs. This is not well-organized, fitness-enhancing behavior! Thus, rather than being an adaptation in its own right, the Cinderella effect in child homicides is best interpreted as a non-adaptive byproduct of discriminative parental solicitude (Daly and Wilson, 1980, 1988), which, like psychological adaptations generally, serves its possessor’s interests on average but not in every instance.

We moved to New Zealand in May, 2019, and with long-standing interests in family violence and substitute parenthood, we were curious to see where our new home stood with respect to these problems. Soon after our arrival, a relevant murder trial was in the news (New Zealand Herald, 2019). On June 11, 2018, a 5-month old infant named Lincoln Wakefield had been shaken so violently that he died of the resultant brain injuries. An autopsy revealed that it was not the first time that he had been shaken with sufficient force to damage his brain. The baby’s mother had been pregnant with Lincoln when she and William met and began dating, and they had set up house together before the birth. Wakefield could work from home, and he encouraged his new partner to return to her job while he cared for the baby. When interviewed by the police on the day of Lincoln’s death, Wakefield eventually confessed to having administered the lethal shaking, explaining his state of mind thus: “I was just gutted he wasn’t mine, to be honest. I just wanted to hurt him until he wasn’t there,” later adding “I was in my own stupid world. I don’t know why I did it. He’s just not mine. It’s hard for me to look at him.” Wakefield denied acting with intent to kill, and offered to plead guilty to manslaughter, but on the day that would have been baby Lincoln’s first birthday, Wakefield was convicted of murder, and given a life sentence. When pronouncing sentence, Justice Dobson addressed the convicted killer, summarizing the picture that he had gained of Wakefield’s mental state in these words: “You tried to deal fondly with Lincoln, but his physical appearance reminded you of his biological father, and increasingly you resented that.” Turning to the court, Justice Dobson added “Baby killings by men who are not the biological fathers of their partners’ children happen far too often in New Zealand.” Sadly, New Zealand is not exceptional in this regard.

Filicides by birth fathers differ from those perpetrated by stepfathers in ways that indicate distinct motives. Daly and Wilson (1994a) reported that over 80% of preschoolers killed by stepfathers in both Canada and Britain died from beatings and/or blunt force trauma, whereas fewer than 50% of the victims of birth fathers were killed in the same way. These results were closely replicated in Weekes-Shackelford and Shackelford’s (2004) analyses of United States data, and clearly imply that impulsive rage reactions are involved in a majority of killings by stepfathers, but in far fewer of those by birth fathers. In all data sets, antipathy toward the child is apparently absent in many birth father cases, and at least a few are misguided “mercy killings,” but in many more cases, filicidal fathers are more depressed than angry. Daly and Wilson (1994a) found that 44% of Canadian men who killed birth children of preschool age committed suicide at the scene of the crime, compared to just 1.5% of those slew stepchildren; directionally similar but less dramatic contrasts were again evident in the British and United States data, too.

These contrasts between filicides perpetrated by stepfathers and those by birth fathers are readily interpreted as indicative of motivational differences whereby stepfathers more often resent the children and their obligations to them. Killings by birth fathers are sometimes brutal, too, but they are proportionately more likely to involve smothering, gassing, and other less assaultive methods. And even when the cases are brutal, filicidal birth fathers rarely if ever offer explanations for their acts like that of another New Zealander who recently killed his 17-month-old stepson when asked to look after him while the mother went out with a girlfriend: In the agreed statement of facts presented at his trial, the killer explained that he was “tired of being treated as the babysitter” (Stuff, 2020). Parental investment is motivated by parents’ intrinsic interest in their children’s wellbeing. Stepparental investment, by contrast, is a courtship gesture and a service offered to the new partner, and when stepparents feel that their contributions are insufficiently appreciated and reciprocated, they are apt to become resentful.

There is a large professional literature, and an even larger self-help literature, dealing with reducing tensions in stepfamilies. One point on which there appears to be near unanimity is that it is a mistake to pretend that a stepfamily is a birth family, or to expect that it will, with time, become psychologically indistinguishable from one (e.g., Johnson, 1980; Turnbull and Turnbull, 1983). The Wakefield case provides a poignant example: Registering the baby’s birth with the stepfather’s surname did not alleviate Wakefield’s distress that Lincoln was “not mine” and may even have exacerbated it.

## Conclusion

It warrants emphasis that stepparent-stepchild relationships need not be toxic, and usually are not. Many children benefit from the presence and involvement of a stepparent (Booth and Dunn, 1994; Thomson et al., 1994) and only occasionally is the average stepchild found to fare worse on some particular measure than the average child raised by a single parent (e.g., McLanahan and Sandefur, 1994; Adjiwanou et al., 2021). Stepparents make valuable investments in stepchildren, even if those investments are restrained relative to those of “natural” (i.e., birth) parents. It is therefore important to investigate what predicts positive stepparental investment, rather than rejection and exploitation, as well as what predicts its variable magnitude. One clearly relevant variable affecting investment by stepfathers is whether they have birth children residing elsewhere (e.g., Hofferth and Anderson, 2003). We suggest that the degree to which their contributions are appreciatively acknowledged may be another, but a good test of this hypothesis will not be easy.

It is often suggested that if a stepfather had been able to bond with his stepchild from birth, he would feel and act like a birth father. The Wakefield case provides reason to doubt this, and so does the only systematic test of which we are aware: In an observational study of men’s interactions with children in a Trinidadian village, Flinn (1988) found not only that stepfathers behaved more “agonistically” toward their partners’ children than did the (presumed) genetic fathers, but also that the several stepfathers who had, like Wakefield, begun cohabiting with women who were already pregnant by other men were significantly *more* hostile toward the resultant stepchildren than other stepfathers, despite having resided with them from birth.

That said, there is evidence that ratings of stepparent-stepchild relationship quality, net of the stepchild’s current age, increase as a function of the relationship’s duration (e.g., Hornstra et al., 2020). To what extent this reflects improvement with time is uncertain, however, since selection (in the sociological rather than Darwinian sense of “selection”) will tend to produce the same pattern, for two reasons. The first is that remarriages with stepchildren are much less stable than first marriages or remarriages without children (e.g., White and Booth, 1985) and marital dissolution is surely selective for conflict-proneness. The second sort of selection occurs when adolescents “vote with their feet”: Stepchildren leave home at earlier average ages than children living with two birth parents (e.g., Kiernan, 1992; Zhao et al., 1995; Davis and Daly, 1997), are massively over-represented among homeless youth (Kufeldt and Nimmo, 1987; Powers et al., 1990), and increasingly have the option of moving to the other birth parent’s home. These selection effects especially challenge interpretation of data from one-off surveys, but they also apply to longitudinal studies with non-negligible drop-out rates since the respondents who are retained may differ systematically from those who drop out.

Rohwer (1986) proposed that the variability among species in their responses to step-offspring (“kill, ignore, or adopt”) can be explained as adaptive responses to ecological and demographic variables that affect the alternatives’ expected fitness effects. Can these principles help explain variability of response within species, too, even within *Homo sapiens*? We have argued that killing stepchildren has clearly not been selected for in the human animal, but must instead be understood as the maladaptive tail of the “rejection” end of an invest-reject distribution, and that non-lethal abuse of a sort that damages the child and thereby raises investment costs is surely maladaptive, too. However, there is a range of lesser forms of maltreatment that may or may not serve the pertrator’s interests, and one might, in principle, propose hypotheses about specific, fine-grained, forms of investment and divestment. We suggest, however, that the adaptations underlying partial investment are probably best characterized at the abstract level of discriminative parental solicitude, and the most promising adaptationist hypotheses for future research are likely to be ones that concern facultative responsiveness to the same ecological and demographic variables that are relevant to the cross-species variability in stepparental response that Rohwer first described and discussed. Candidate variables include cues of local sex ratios and of the local levels of marital stability, mortality, requisite resources (e.g., Willführ and Gagnon, 2013). and environmental predictability, as well as the reproductive values of both mates, child attributes, and whether and to what degree investing in the children will diminish or enhance those values. In short, attention to theory and research on sexual selection promises to inspire and inform future studies of stepfamily dynamics.

## Author Contributions

MD wrote the manuscript and GP edited it. Both authors conceived the manuscript together, contributed to the article, and approved the submitted version.

## Conflict of Interest

The authors declare that the research was conducted in the absence of any commercial or financial relationships that could be construed as a potential conflict of interest.

## Publisher’s Note

All claims expressed in this article are solely those of the authors and do not necessarily represent those of their affiliated organizations, or those of the publisher, the editors and the reviewers. Any product that may be evaluated in this article, or claim that may be made by its manufacturer, is not guaranteed or endorsed by the publisher.
